# NF2 alteration in mesothelioma

**DOI:** 10.3389/ftox.2023.1161995

**Published:** 2023-04-25

**Authors:** Yoshitaka Sekido, Tatsuhiro Sato

**Affiliations:** ^1^ Division of Cancer Biology, Aichi Cancer Center Research Institute, Nagoya, Japan; ^2^ Division of Molecular and Cellular Oncology, Nagoya University Graduate School of Medicine, Nagoya, Japan

**Keywords:** mesothelioma, genome, tumor suppressor gene, *NF2*, Hippo pathway

## Abstract

The *NF2* tumor suppressor gene is a frequent somatically mutated gene in mesothelioma, with 30%–40% mesotheliomas showing *NF2* inactivation. *NF2* encodes merlin, a member of the ezrin, radixin, and moesin (ERM) family of proteins that regulate cytoskeleton and cell signaling. Recent genome analysis revealed that *NF2* alteration may be a late event in mesothelioma development, suggesting that *NF2* mutation confers a more aggressive phenotype to mesothelioma cells and may not be directly caused by asbestos exposure. The Hippo tumor-suppressive and mTOR prooncogenic signaling pathways are crucial cell-signaling cascades regulated by merlin. Although the exact role and timing of *NF2* inactivation in mesothelioma cells remain to be elucidated, targeting the *NF2*/merlin-Hippo pathway may be a new therapeutic strategy for patients with mesothelioma.

## 1 Introduction

Mesothelioma shows frequent somatic alterations in a few tumor suppressor genes, such as *BAP1*, *NF2*, *CDKN2A/2B*, and *TP53*. Other infrequently but characteristically mutated genes identified in mesothelioma are those involved in histone modifications and RNA processing (Bueno et al., 2016; Hmeljak et al., 2018). While oncogenic driver mutations of tyrosine kinases are rarely detected in mesothelioma, a promoter mutation in *TERT* that is essential for telomerase activity is frequently observed, especially in non-epithelioid histology (Tallet et al., 2014; Pirker et al., 2020). Chromosomal abnormalities in mesothelioma are characterized by frequent chromosomal loss, with some showing extensive chromosome loss, termed “genomic near-haploidization” (Hmeljak et al., 2018). Chromosomal rearrangements, including chromoplexy and chromothripsis, have also been detected in the mesothelioma genome, possibly resulting in the potential for neoantigen presentation (Mansfield et al., 2020). A recent comprehensive analysis using multiple sampling detected subclonal populations in mesothelioma tissues, suggesting that some genomic changes are early events and others are late (Meiller et al., 2021; Zhang et al., 2021). Among them, *NF2* mutation seems to be a late event, indicating that the direct asbestos effect might not be related to the genomic damage of *NF2* (Meiller et al., 2021; Zhang et al., 2021). Nonetheless, targeting the Hippo signaling pathway, which is regulated by merlin (*NF2* product), is a promising therapeutic modality for developing inhibitors against transcriptional enhanced associated domain (TEAD) transcriptional factors. Thus, while the roles and timing of *NF2* inactivation in mesothelioma cells remain to be fully elucidated, the study of *NF2* in mesothelioma is a focus of much research to develop new diagnostic and therapeutic tools against this formidable disease.

## 2 *NF2*

**FIGURE 1:**
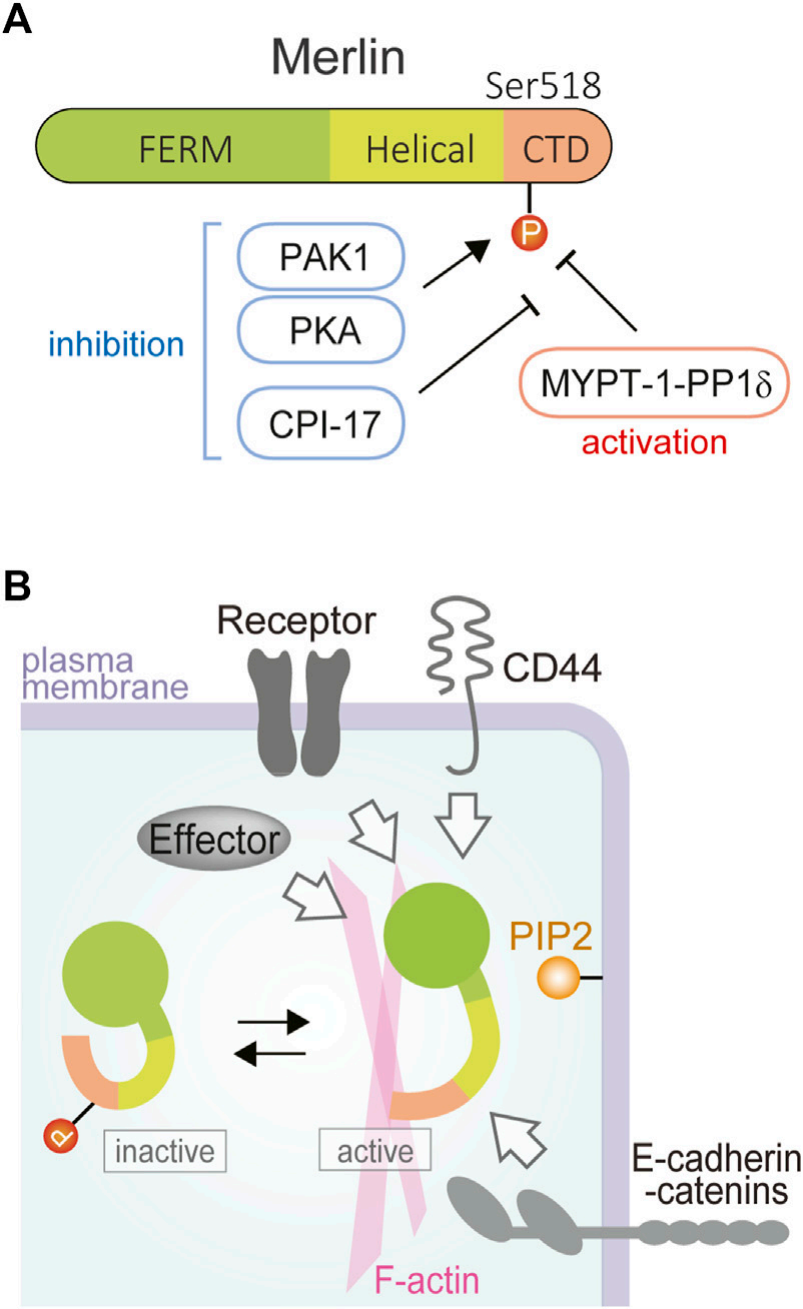
Domain structure and activation-inactivation of merlin. **(A)** Merlin is composed of N-terminal FERM (4.1/ezrin/radixin/moesin) domain (green), a central alpha-helical domain (light green), and a C-terminal domain (CTD; orange). Ser518 is one of the major phosphorylation sites, phosphorylated with PAK or PKA and dephosphorylated with myosin phosphatase holoenzyme MYPT-1-PP1δ that is encoded by *PPP1R12A* for the major regulatory subunit MYPT-1 and by *PPP1CB* for the catalytic subunit PP1β/δ. **(B)** Merlin activation is regulated by many membrane-associated proteins such as receptor tyrosine kinases, CD44, and E-cadherin-alpha catenin. Merlin activation is thought to be induced by dephosphorylation and lipid binding. Merlin interacts with the plasma membrane and the cortical actin skeleton to exert tumor-suppressive activity.

*NF2* was originally discovered to be responsible for a familial cancer syndrome, neurofibromatosis type II, and is located in chromosome 22q12 (Rouleau et al., 1993; Trofatter et al., 1993). The protein encoded by *NF2* is moesin-ezrin-radixin-like protein (merlin, also known as neurofibromin 2 or schwannomin), a member of the Band 4.1 family of cytoskeletal linker proteins (Petrilli and Fernandez-Valle, 2016). Merlin is a 70-kDa protein with three distinct domains, consisting of the FERM (4.1, ezrin, radixin, and moesin) domain at the N-terminus, an alpha-helical domain and a C-terminal domain (Figure 1A). In contrast to other ezrin, radixin, and moesin (ERM) family of proteins, merlin does not have an actin-binding site in the C-terminal domain but instead has a unique actin-binding motif in the N-terminal domain.

The tumor suppressive activity of merlin is regulated by conformational changes in the merlin molecule, which are due to the head-tail interaction of the merlin molecule, such as by phosphorylation modification (Figures 1A, B). One specific mechanism to inactivate merlin is thought to be the phosphorylation of the S518 residue in the tail domain by protein kinase A (PKA) or p21-activated kinase (PAK), while myosin phosphatase MYPT1-PP1δ activates merlin by dephosphorylating S518. Whether the open or closed form of merlin acts as a functional tumor suppressor is still unclear, but a structural study suggested that lipid binding of unphosphorylated merlin causes the open conformation of merlin and activates its tumor-suppressor function (Chinthalapudi et al., 2018).

Merlin indirectly links F-actin, transmembrane proteins, and intracellular effectors as a scaffold protein to regulate intracellular pathways that control cell survival and proliferation (McClatchey and Giovannini, 2005; Curto and McClatchey, 2008). These molecules include cell adhesion molecules and receptor tyrosine kinases (RTKs) that receive extracellular conditions/signals and downstream molecules associated with intracellular signal transduction cascades such as phosphoinositide 3-kinase (PI3K)/Akt, Hippo, and mammalian target of rapamycin (mTOR) pathways, which are prooncogenic or tumor suppressive pathways that have extensively been studied (Sato and Sekido, 2018).

Clinically, carriers with *NF2* germline mutations are predisposed to bilateral vestibular schwannomas, those of other cranial and peripheral nerves, meningiomas, and ependymomas (Ferner, 2007). Interestingly, patients and carriers of *NF2* familial cancer syndrome do not seem to present a significantly increased risk of mesothelioma (Asthagiri et al., 2009), and only a few rare cases of mesothelioma development with constitutional *NF2* mutation have been reported so far (Baser et al., 2005). Patients with *NF2* germline mutation have very different prognoses depending on ages of onset, tumor types, and types of genetic mutation; in a 30-year follow-up study of 353 patients, those observed to be symptomatic at age ≤25 had relatively poor prognoses, with about 10% dying 15 years after diagnosis, and the average life expectancy of those who died was in the 30s (Forde et al., 2021). Meanwhile, only about 10% of *NF2* patients died within 15 years of diagnosis, and many survived for a long time (Forde et al., 2021). Therefore, although many *NF2* patients reach the age of morbidity for mesothelioma onset, the development of mesothelioma has not specifically been reported, suggesting that *NF2* germ line mutation is unlikely to be a predisposing mutation for familial mesothelioma development like *BAP1*.

*NF2* somatic mutations have also been observed in sporadic tumors related to *NF2* cancer syndrome such as meningioma (Nassiri et al., 2021), vestibular schwannoma (Carlson et al., 2018), and other malignancies, albeit at low frequency, including breast (Lang et al., 2020), liver (Zhang et al., 2017), and kidney (Hacking et al., 2023) cancers.

## 3 *NF2* alteration in mesothelioma

Somatic mutation of *NF2* is harbored in 30%–40% of pleural mesotheliomas (Bueno et al., 2016; Hmeljak et al., 2018; Sato and Sekido, 2018; Hiltbrunner et al., 2022). Peritoneal mesothelioma are 10%–20% of all mesothelioma cases, and *NF2* mutation is seen in 21%–35% of cases (Hung et al., 2020; Hiltbrunner et al., 2022; Offin et al., 2022). In addition to non-sense/missense mutations or small/large deletions with loss of heterozygosity, resulting in bi-allelic loss of function, other structural abnormalities including gene rearrangement that disrupt the *NF2* region can be observed in mesotheliomas (Bueno et al., 2016; Hmeljak et al., 2018). Noticeably, *NF2* mutations are found more frequently in sarcomatoid rather than in epithelioid mesothelioma (Quetel et al., 2020).

Recent genome analysis with multi-sampling of the same patient’s mesothelioma tissues revealed clonality of mesothelioma cells, which showed that *BAP1*/3p21 loss is an early event, while *NF2*/22q loss is a late event (Zhang et al., 2021). Intra-tumor heterogeneity of *NF2* mutation in pleural mesothelioma has also been observed in another study, indicating that *NF2* mutation is a late event that may result in more aggressive phenotypes (Meiller et al., 2021).

**FIGURE 2:**
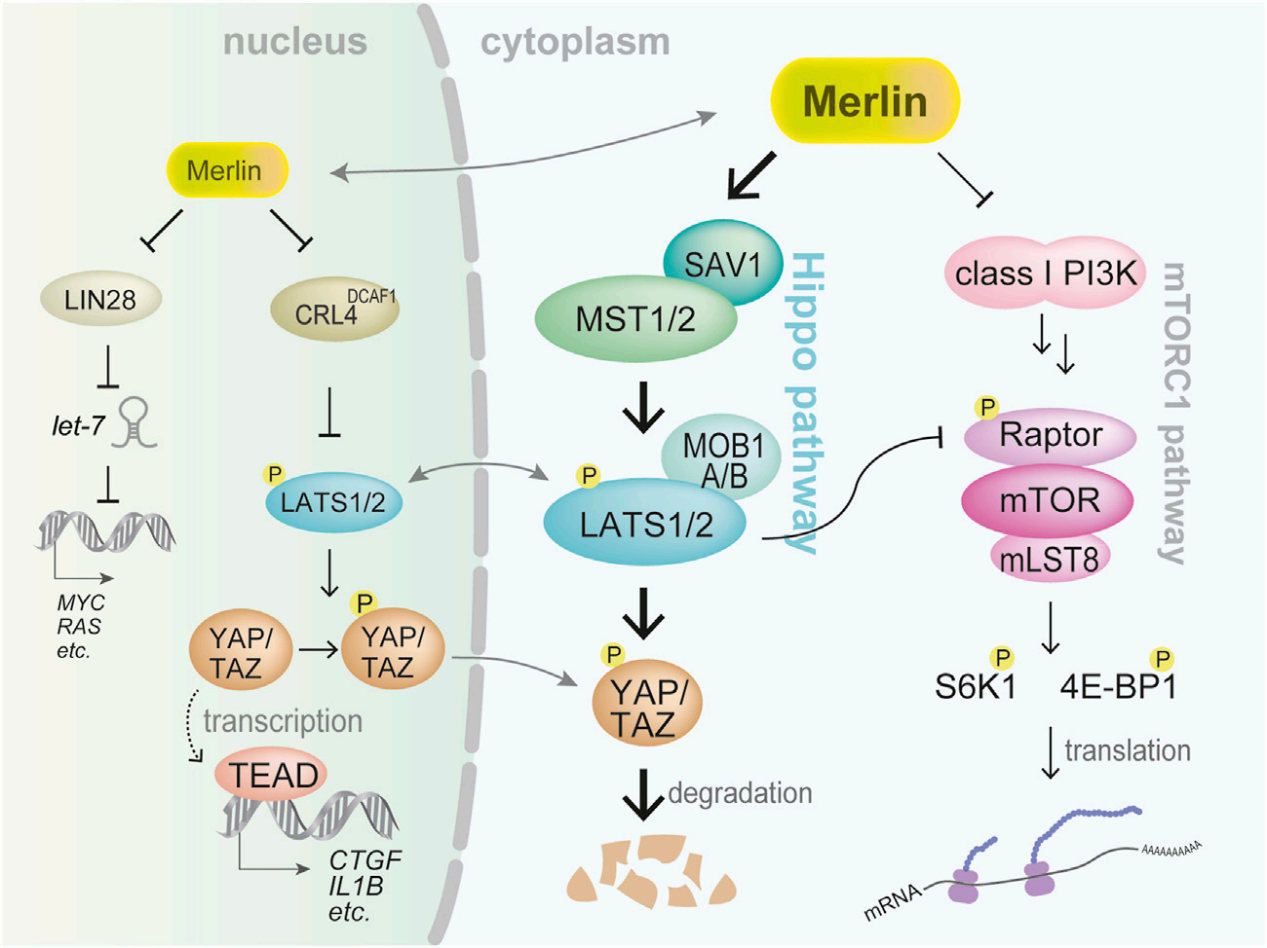
Representative signaling pathways regulated by merlin. Two significant downstream signaling pathways mediated through the cytoplasm are the Hippo and mTORC1 pathways. Under merlin inactivation, underphosphorylated (active) YAP/TAZ transcriptional coactivators bind to TEAD family transcription factor resulting in the expression of multiple prooncogenic genes such as *CTGF* and *IL1B*. Merlin also regulates mTORC1 activation that enhances translation of proteins. Merlin is also thought to translocate into the nucleus to exert its tumor-suppressive function.

Besides *NF2*, mesotheliomas harbor frequent inactivation of genes of the Hippo pathway, which seems to be an important signaling pathway regulated by merlin in mesothelial cells (Figure 2). *LATS2* genetic alterations have been observed in 7%–11% of mesothelioma cases (Murakami et al., 2011; Bueno et al., 2016; Tranchant et al., 2017; Hmeljak et al., 2018). A comprehensive genome analysis of mesothelioma tissues detected frequent allelic loss among several Hippo pathway gene regions such as *MST1* and *LATS1* (Bueno et al., 2016). Epigenetic alterations in the promoter sequences of these component genes have also been detected (Hmeljak et al., 2018).

These results suggest that the overall frequency of genetic abnormalities in *NF2* and the Hippo pathway component genes may be underestimated and slightly higher than those previously reported in mesothelioma tissues. Regarding *NF2*, whole-exome sequencing alone cannot detect translocation and large deletion of *NF2*, which occasionally happens in mesothelioma. Even point mutations of *NF2* might have been evaluated as wild-type in the samples analyzed, because in the above studies examining multiple specimens, 22% (Zhang et al., 2021) and 38% (Meiller et al., 2021) of the *NF2* mutations detected were subclonal. Therefore, despite the presence of *NF2* alterations, it is possible that *NF2* gene abnormalities were overlooked depending on the location of the tumor tissue analyzed. A more important implication may be that *NF2* mutations are not caused by a direct inflammatory response or DNA damage induced by asbestos. This idea is consistent with the absence of reports of increased risk of mesothelioma in *NF2* germline mutation carriers.

In addition to the genetic or epigenetic inactivation of *NF2* itself, other mechanisms are also known to be possibly involved in merlin inactivation. While MYPT-1-PP1δ (encoded by *PPP1R12A* and *PPP1CB*) dephosphorylates (activates) merlin, protein kinase C-potentiated phosphatase inhibitor of 17 kDa (CPI-17) (encoded by *PPP1R14A*) inhibits MYPT-1-PP1δ (Figure 1A) (Jin et al., 2006). Accordingly, merlin tends to be phosphorylated (inactivated) by CPI-17 overexpression, which has been demonstrated in mesothelioma tissues (Thurneysen et al., 2009). *NF2* mRNA was reported to have splicing variants, and the same study indicated that the carboxyl-terminal variant (isoform 2), which may not have tumor suppressive activity, is more significantly expressed in mesothelioma (Thurneysen et al., 2009). *NF2* also seemed to be suppressed by overexpression of specific *NF2*-targeting microRNAs including has-miR-885-3p (Guled et al., 2009).

## 4 Tumor-suppressive activity of *NF2* gene in mesothelioma cells

*In vitro* transduction of *NF2* can suppress proliferation and other malignant phenotypes of *NF2*-deficient mesothelioma cells (Xiao et al., 2005). In mesothelioma cells, merlin is thought to function as a tumor suppressor through multiple mechanisms. An early study indicated that merlin inhibits focal adhesion kinase (FAK), resulting in the inhibition of the interaction between FAK and its binding partners Src and p85, the regulatory subunit of PI3K (Poulikakos et al., 2006). Thus, one of the prooncogenic effects of *NF2* inactivation may be accompanied with the upregulation of FAK activity.

The significance of *Nf2* inactivation in mesothelioma pathogenesis was confirmed in *Nf2*-knockout mouse models *in vivo*. For instance, *Nf2* (+/−) knockout mice showed accelerated mesothelioma tumor formation following asbestos exposure compared to the wild-type littermates with asbestos exposure only (Altomare et al., 2005). Conditional knockout mice targeting *Nf2*, *Bap1*, and/or *Cdkn2a* in cells of the mesothelium exhibited an increased incidence of mesothelioma, and noticeably, the triple-knockout mice showed highly invasive tumors with the shortest survival times (Kukuyan et al., 2019). Similar triple-knockout mouse models also confirmed that, although *Bap1* deletion alone did not induce mesothelioma, *Bap1* deletion dramatically accelerated mesothelioma development with the combined disruption of *Nf2* and *Cdkn2ab* (Badhai et al., 2020).

Notably, in addition to functioning under the cytoplasmic membrane, merlin is thought to translocate into the cell nucleus (Figure 2). Merlin was shown to bind to CRL4^DCAF1^ and E3 ubiquitin ligase, thereby inhibiting the function of CRL4^DCAF1^ in the ubiquitination of its target protein (Li et al., 2010). Thus, merlin acted as a tumor suppressor by inhibiting CRL4^DCAF1^ in mesothelioma cells (Li et al., 2010). Moreover, CRL4^DCAF1^ was shown to directly bind to LATS1/2 and induce their ubiquitination and degradation (Li et al., 2014); thus, upregulated CRL4^DCAF1^ in *NF2*-inactivated mesothelioma cells accelerates degradation of LATS1/2 and hence activates YAP.

Independent of the Hippo pathway, merlin can exhibit cell-density-dependent, tumor-suppressive activity by inhibiting Lin28B function and promoting *let-7* microRNA biogenesis (Hikasa et al., 2016). Since *let-7* miRNAs silence *RAS* and *MYC*, the downregulation of *let-7* by Lin28B induces mesothelioma cell proliferation.

## 5 Hippo pathway inactivation and targeting drugs

Hippo pathway is a significant signaling pathway regulated by merlin (Figure 2). The Hippo pathway participates in various physiological processes, including cell proliferation, cell differentiation, organ growth, embryogenesis, tissue regeneration, and wound healing (Fu et al., 2022). Dysregulation of this pathway is associated with various diseases including cardiac disease, pulmonary disease, hepatic disease, renal disease, and cancer (Fu et al., 2022). Hippo pathway composed of many molecules and classical core components are MST1 and MST2 kinases, SAV1 (also termed WW45), MOB1A/B, and LATS1 and LATS2 kinases, and a variety of signals and extracellular conditions including cell density, cell polarity, cell attachment, mechanical cues, and soluble factors, regulate the Hippo pathway (Fu et al., 2022).

Under the Hippo pathway activation, MST1 and MST2 kinases phosphorylate (activate) LATS1 and LATS2. MAP4Ks can also phosphorylate LATS1/2. Major targets of LATS1/2 kinases are YAP and TAZ transcriptional coactivators. When YAP/TAZ are phosphorylated, they are either retained within the cytoplasm or degraded. Conversely, when the Hippo pathway is inactivated, under-phosphorylated YAP/TAZ enters the cell nucleus and acts as a transcriptional coactivator. YAP/TAZ binds to distinct types of transcription factors including the TEAD family (Currey et al., 2021). Exogenously transduced constitutively activated mutant *YAP S127A* or *TAZ S89A* was shown to induce immortalized human mesothelial cells to form mesothelioma-like tumors in xenograft models (Kakiuchi et al., 2016; Matsushita et al., 2019).

In mesothelioma cells, activated YAP/TAZ enhances the transcription of a several important genes to stimulate cancer-promotion and progression, such as those encoding connective tissue growth factor (*CTGF*) (Fujii et al., 2012), phospholipase-C beta 4 (*PLCB4*) (Kakiuchi et al., 2016), and interleukin 1β (*IL1B*) (Matsushita et al., 2019). CTGF is an extracellular matrix-associated matricellular protein, and its overexpression appears to induce abundant extracellular matrix formation in mesothelioma (Fujii et al., 2012). Besides Hippo inactivation, TGF-β stimulation (Fujii et al., 2012) and β-catenin-TCF-LEF signaling (Jiang et al., 2014) further enhance the expression of CTGF. CTGF expression is higher in the sarcomatoid than in epithelioid mesothelioma, and it mediates the epithelial-mesenchymal transition (EMT) in mesothelioma (Jiang et al., 2014).

Since YAP/TAZ have been recognized as unlikely druggable targets, molecules that block the interaction of YAP/TAZ with TEADs have been vigorously investigated. Verteporfin (Visudyne) was the first small molecule that inhibited YAP-TEAD binding (Liu-Chittenden et al., 2012). Verteporfin suppresses YAP activity along with malignant phenotypes of mesothelioma cells *in vitro*, but the exact mechanisms by which verteporfin targets YAP are not fully understood (Tranchant et al., 2017). Recently, several groups have developed small molecules to inhibit the auto-palmitoylation of TEAD (Kaneda et al., 2020; Tang et al., 2021). These molecules share a common mechanism of binding to a conserved Cys residue (e.g., Cys359 of TEAD1), a palmitoylation site critical for YAP/TAZ and TEAD interaction, thus inhibiting YAP/TAZ binding to TEAD. These TEAD inhibitors have been shown to suppress tumors *in vivo* models (Kaneda et al., 2020; Tang et al., 2021). Although some molecules are under clinical study (Zagiel et al., 2022), TEAD palmitoylation inhibitors have also been implicated to have some therapeutic limitations in cancer cells (Sun et al., 2022).

## 6 Other therapeutic strategies for *NF2*-deficient mesothelioma

The merlin-Hippo pathway influences the activation of the PI3K-AKT-mTOR pathway. For example, merlin binds to PI3K enhancer-L and inhibits PI3K (Rong et al., 2004) (Figure 2). Furthermore, LATS1/2 inhibits mTORC1 activation by phosphorylating Raptor (Gan et al., 2020) (Figure 2). In addition, YAP induces AKT activation by inducing miR-29 expression and suppressing *PTEN* translation (Tumaneng et al., 2012). Therefore, molecules in the merlin-Hippo pathway influence mTORC1 activation at various levels.

To date, mTOR inhibitors have been investigated, and an allosteric mTOR inhibitor, rapamycin, has been demonstrated to have selective growth inhibitory effects in *NF2*-deficient mesothelioma cells (Lopez-Lago et al., 2009). However, a phase-II study of the mTOR inhibitor everolimus showed its limited clinical activity in advanced pleural mesothelioma (Ou et al., 2015). Meanwhile, a phase-I study using the class I PI3K and mTOR kinase dual inhibitor GDC-0980 (apitolisib) demonstrated partial response in pleural and peritoneal mesothelioma patients (Dolly et al., 2016). When considering what type of mTOR inhibitor to develop in future, it might be necessary to consider the influence of activation/inactivation of molecules of the merlin-Hippo pathway.

Loss of *NF2* leads to PAK activation (Kissil et al., 2003). Thus, PAK inhibitors have been considered as for the treatment of *NF2*-deficient tumors. A recent study indicated that *Nf2*; *Cdkn2a*-deficient mouse mesothelioma cells showed less malignant phenotypes with *PAK2* loss, suggesting that anti-PAK drugs may be promising for mesothelioma treatment (Sementino et al., 2022).

Another promising therapeutic strategy is the induction of synthetic lethality in mesothelioma cells. For instance, focal adhesion kinase (FAK) inhibition shows synthetic lethality with *NF2* loss. VS-4718, a FAK inhibitor, suppressed proliferation and induced apoptosis in merlin-negative mesothelioma cells (Shapiro et al., 2014). However, a phase-II study using another FAK inhibitor, VS-6063 (defactinib), did not confirm clinical benefits as a maintenance therapy for pleural mesothelioma (Fennell et al., 2019). In this regard, E-cadherin expression was shown to be associated with the resistance of *NF2*-deficient mesothelioma cells to VS-4718, presenting a possible reason for why the clinical study did not show benefits (Kato et al., 2017). Another study showed that defactinib sensitivity using mesothelioma cell lines was not related to inactivating *NF2* mutations or protein expression of merlin, but was strongly correlated with FAK activity, especially in epithelioid mesothelioma (Tranchant et al., 2018). Therefore, the hypothesis that FAK inhibitors exhibit synthetic lethality with *NF2* mutations is not fully confirmed; however, its biomarker needs to be identified since FAK inhibitors are effective in a subset of mesothelioma cells with high FAK activity, and the study has identified *LUM*, a regulator of the integrin pathway, as a candidate (Tranchant et al., 2018).

Meanwhile, for *NF2*-mutated meningiomas, a phase-II study of GSK2256098, another FAK inhibitor, has been shown to be well tolerated, resulting in an improved progression-free survival rate (Brastianos et al., 2022). In addition to FAK, the discovery of new molecules or drugs that can induce synthetic lethal phonotype with *NF2* deficiency may provide a new therapeutical strategy for mesothelioma.

## 7 Ferroptosis induction by YAP

From their discovery, YAP/TAZ have been thought to act as both oncoproteins and tumor-suppressive proteins, depending on the cellular context. The tumor suppressive activities of YAP/TAZ have been reported in the colon, estrogen receptor-positive breast, hematological, and other human solid cancers (Pearson et al., 2021). Ferroptosis refers to regulated cell death that is characterized by the iron-dependent accumulation of lipid hydorperoxides to lethal levels (Stockwell et al., 2017). A central regulator of ferroptosis is glutathione peroxidase 4 (GPX4), which protects cells by eliminating lipid peroxides, and cancer cells with mesenchymal or metastatic property are highly susceptible to ferroptosis (Hangauer et al., 2017; Viswanathan et al., 2017). Notably, ferroptosis was shown to be regulated by cadherin-mediated intercellular interactions and the merlin (*NF2*)-Hippo pathway (Wu et al., 2019). *NF2* inactivation sensitized cancer cells to ferroptosis because YAP induced the expression of the crucial mediators of ferroptosis such as transferrin receptor 1 (*TFRC*) and acyl-CoA synthetase long chain family member 4 (*ACSL4*), suggesting that YAP can act as a tumor suppressor dictating ferroptotic death including mesothelioma (Wu et al., 2019). Thus, the inactivation of merlin (*NF2*)-Hippo pathway status has been proposed as a predictor of the sensitiveness of cancer cells to ferroptosis-induction therapies (Wu et al., 2019).

The results are contrary to previous findings that YAP activation in mesothelioma cells indicates oncogenic function. One explanation for the result might be that YAP activation is affected by various factors, including the extracellular environment and intracellular signaling/metabolism; therefore, changes in such conditions can alter the gene transcription pattern regulated by YAP, resulting in the predominant expression of a group of cancer-promoting genes or, if the conditions are met, those of tumor suppressive genes, including ferroptosis-related genes. Furthermore, loss of function of *p53* (Jiang et al., 2015) and *BAP1* (Zhang et al., 2018) has been reported to enhance resistance to ferroptosis, and it is possible that YAP functions more in a cancer-promoting manner in mesothelioma cells with those mutations, although further detailed studies are required.

## 8 *NF2* mutation for mesothelioma diagnosis

*NF2* deficiency has been shown to have utility as a diagnostic, prognostic, and/or predictive biomarker for mesothelioma. Genetic testing for *CDKN2A/2B* loss and immunohistochemistry (IHC) for methylthioadenosine phosphorylase (MTAP) loss and BAP1 loss are currently thought to be the most reliable molecular tools for differentiating mesothelioma from other malignancies and, often most critically, from reactive mesothelial hyperplasia, which is a benign change. A recent study indicated that the addition of genetic *NF2* screening improved the sensitivity or specificity of mesothelioma diagnosis (Nabeshima et al., 2022). Another study reported that low expression of merlin and high labeling index of survivin were correlated with poor prognosis in pleural mesothelioma patients (Meerang et al., 2016). Similarly, a combination of *CDKN2A* homozygous deletion and *NF2* hemizygous loss is a negative prognostic factor for patients with peritoneal mesothelioma (Singhi et al., 2016). Regarding the IHC study of merlin, which has been recognized as challenging with currently available antibodies for merlin, a recent study indicated that the expression of merlin was absent in 52% of all pleural mesotheliomas and 70% of non-epithelioid tumors (Chapel et al., 2022).

## 9 Conclusion

*NF2* alteration is a key genetic change in mesothelioma development, which may occur at a late stage as a subclonal event, suggesting that *NF2* alteration may not be directly induced by asbestos exposure. *NF2* mutation is thus thought to confer a more aggressive phenotype to mesothelioma cells, possibly involved in the induction of EMT. The Hippo pathway is a crucial signaling cascade that is regulated by merlin (*NF2*), which is currently considered a promising target for patients with mesothelioma. Future studies are needed to more precisely identify the roles of merlin (*NF2*) alteration in mesothelioma development and progression, which would lead to the development of new molecular-targeted drugs.
